# A novel hybrid ultrafast shape descriptor method for use in virtual screening

**DOI:** 10.1186/1752-153X-2-3

**Published:** 2008-02-18

**Authors:** Edward O Cannon, Florian Nigsch, John BO Mitchell

**Affiliations:** 1 Unilever Centre for Molecular Science Informatics, Department of Chemistry, University of Cambridge, Lensfield Road, Cambridge, CB2 1EW, UK

## Abstract

**Background:**

We have introduced a new Hybrid descriptor composed of the MACCS key descriptor encoding topological information and Ballester and Richards' Ultrafast Shape Recognition (USR) descriptor. The latter one is calculated from the moments of the distribution of the interatomic distances, and in this work we also included higher moments than in the original implementation.

**Results:**

The performance of this Hybrid descriptor is assessed using Random Forest and a dataset of 116,476 molecules. Our dataset includes 5,245 molecules in ten classes from the 2005 World Anti-Doping Agency (WADA) dataset and 111,231 molecules from the National Cancer Institute (NCI) database. In a 10-fold Monte Carlo cross-validation this dataset was partitioned into three distinct parts for training, optimisation of an internal threshold that we introduced, and validation of the resulting model. The standard errors obtained were used to assess statistical significance of observed improvements in performance of our new descriptor.

**Conclusion:**

The Hybrid descriptor was compared to the MACCS key descriptor, USR with the first three (USR), four (UF4) and five (UF5) moments, and a combination of MACCS with USR (three moments). The MACCS key descriptor was not combined with UF5, due to similar performance of UF5 and UF4. Superior performance in terms of all figures of merit was found for the MACCS/UF4 Hybrid descriptor with respect to all other descriptors examined. These figures of merit include recall in the top 1% and top 5% of the ranked validation sets, precision, F-measure, area under the Receiver Operating Characteristic curve and Matthews Correlation Coefficient.

## Background

The use of illegal performance enhancing substances continues to threaten both the integrity of sporting competition and the health of athletes. The World Anti-Doping Agency (WADA) [1] was established in 1999 as an independent and international anti-doping body. Its mission is to combat doping in sport on a worldwide scale. Over the nine years since WADA was established, a large amount of investment has gone into research. More than 60 research proposals were received and 22 were selected for funding by WADA in 2005. In 2006, 71 applications were received for research proposals, of which 25 were selected. WADA has invested a grand total of US$28m into research from the year 2000. We are aware of only three applications of chemoinformatics to prohibited substances [2-4]. All the chemoinformatics methods to date have used machine learning methods to classify prohibited substances into their respective categories using chemical descriptors calculated by computers or data collated from analytical laboratory instruments. An alternative approach is to use a machine learning algorithm to virtually screen a database of compounds. Here the objective is to rank molecules based on their probability of being active relative to a reference structure or set of structures and this method has been used in the past by the pharmaceutical industry to search for novel lead compounds and identify compounds potentially appropriate for a given receptor. There are a number of different methods that can be used for virtual screening of databases of chemical compounds. However, this work involves similarity-based virtual screening.

Similarity-based virtual screening is founded on the similar property principle [5], which states that molecules with similar structures often exhibit similar properties and biological activity. Similarity-based virtual screening is a technique used to rank the compounds of large databases based on how similar they are to one, or several, reference molecules of pharmacological interest. The rank assigned to a molecule reflects its probability of being active. Molecules in the top few ranks of a sorted database are expected to be very similar to the biologically active query molecules in the training set and are thus assigned a higher probability of being active. One of the main applications of similarity-based virtual screening is to help decide which compounds should be taken forward for *in vitro *screening. Other related applications include identifying which compounds should be purchased from an external vendor or which libraries to synthesise [6].

There are a number of important steps in conducting a similarity-based virtual screening experiment, the first of which is to define the representation of the molecules in chemical space using descriptors. Descriptors are usually defined by their dimensionality. One-dimensional descriptors are properties such as molecular weight and log P [7]. Two-dimensional descriptors are derived from the connection table [8], whilst three-dimensional descriptors use geometric information from molecular structures in three-dimensional space [9]. Probably the most commonly used descriptors are those based on two-dimensional structure [6,10]. Such descriptors are usually binary in nature and typically encode the presence or absence of substructural fragments, a prime example being the MACCS key descriptor [11]. Hashed fingerprints are also commonly used, and differ from structural key descriptors in that they do not use a predefined dictionary, but incorporate patterns, often made up of atom types, augmented atoms and atom paths. The Daylight fingerprint [12] of length 1024 bits is an example of a hashed fingerprint.

Such descriptors have been reported by Hert et al. [13,14] and Bender et al. [15] to perform well in the domain of similarity-based virtual screening. In contrast, until fairly recently, it has been accepted that three dimensional methods do not perform as well as existing two dimensional methods solely in terms of the number of actives retrieved [16], if the actives have a larger common substructure to each other than to the negatives [17]. Most three dimensional methods have performed less well in the past due to the fact that three dimensional descriptors have to deal with translational and rotational variance in addition to a potentially large number of conformations.

Previous studies have shown that one of the key features in discriminating active from inactive molecules is molecular shape [18,19]. However, as with many other three dimensional methods, it is often said that the problem of calculating molecular shape is not only a very challenging task, it is also time consuming. A recent shape descriptor proposed by Ballester and Richards [20] called Ultrafast Shape Recognition (USR) has been shown to avoid the alignment problem, and to be up to 1500 times faster to calculate than other current methodologies. The shape descriptor makes the assumption that a molecule's shape can be uniquely defined by the relative position of its atoms and that three-dimensional shape can be characterised by one-dimensional distributions. They compared the performance of USR to the EigenSpectrum Shape Fingerprints (EShape3D) in the Molecular Operating Environment (MOE) [21] by visualising the shapes of the top few hits in the ranked database and found that similar shapes were retrieved for both methods.

Recent work by Baber et al. [22] has shown that combining two and three-dimensional methods can improve virtual screening performance. Baber et al. have used consensus scoring in ligand based virtual screening with a set of two and three dimensional structural and pharmacophore based descriptors and found that consensus scores generally worked better than single scores. The improvement in performance was attributed to additional information relevant to ligand – receptor binding.

The second stage in any virtual screen is to decide the number of bioactive reference compounds. Past virtual screening studies have mainly been concerned with the use of a single bioactive reference structure. However more recent studies have used multiple bioactive references [13]. A common way to rank molecules is to select a training set of actives and inactives for the training of a classification method in order to predict the likelihood of unseen molecules in a test set being active; the molecules are then ranked based on this likelihood. A number of machine learning methods have been used: support vector machine [23], *k*-nearest neighbour [24], binary kernel discrimination [13], neural networks [25] and the naive Bayes classifier [15]. More recently, the Random Forest classification algorithm has successfully been used to screen a database of ~8,000 Chinese herbal substances for potential inhibitors of several therapeutically important molecular targets [26].

The final stage is to assess the performance of the descriptor and classification method. Common methods used in the machine learning and information retrieval community are: recall, precision, the F-measure, Matthews Correlation Coefficient and the area under the Receiver Operating Characteristic curve (AUC).

In this paper, we report the use of a novel Hybrid descriptor that combines both two and three-dimensional information. The novel descriptor is composed of the MACCS key 166 bit packed descriptor, which is binary in nature (composed of 0s and 1s) and the USR descriptor which is based on 12 floating point numbers. We have extended the USR descriptor to include 4 additional floating point numbers from the calculation of the fourth moment (kurtosis) of the interatomic distance distributions. Concatenated together, this makes a descriptor of 182 components.

0101110011..._*MACCS*(166) _+ 2.567..._*USR*(16)_

We have used the Random Forest classifier to conduct a virtual screen and rank molecules taken from the WADA 2005 dataset and the National Cancer Institute (NCI) database based on their probability of being active. We have assessed the Hybrid descriptor's performance against the USR descriptor (with three moments), the USR descriptor with four and five moments (UF4, UF5) and the MACCS key descriptor on an external validation set and report: the recall of actives in the top 1% and top 5% of the validation sets, precision, the F-measure, Matthews Correlation Coefficient and the area under the Receiver Operating Characteristic curve of the ranked validation sets. Details of the performance measures can be found below.

## Methods

### Dataset and preprocessing

All 249,071 three dimensional CORINA[27] generated structures were taken from the publicly available 1999 National Cancer Institute database [28]. Duplicates were removed, leaving 236,936 unique structures, which were then filtered for drug-likeness, using a Lipinski filter [29] in MOE [21], leaving 111,694 structures. A further 463 metal ion complexes were filtered from the database as no bits were set in the MACCS key descriptor for these compounds. This left a dataset of 111,231 NCI compounds. These compounds were combined with the original 5,245 compounds taken from the 2005 WADA Anti-Doping Agency (WADA) dataset [1] to form a final dataset of 116,476 compounds. The WADA classes are composed of molecules explicitly on the prohibited list and of molecules of similar biological activity and chemical structure taken from the MDDR database [11] (version 2003.1). The WADA dataset is composed of 10 different activity classes: beta blockers (P2), anabolic agents (S1), hormones and related substances (S2), β-2 agonists (S3), agents with anti-estrogenic activity (S4), diuretics and other masking agents (S5), stimulants (S6), narcotics (S7), cannabinoids (S8) and glucocorticosteroids (S9). The breakdown of the WADA activity classes is given in Table 1 and in the supplementary information (Additional file 1). Pictures of the most and least representative molecules for each prohibited class can also be found in the supplementary information (see Additional files 2 and 3). For the purpose of this work, the NCI compounds were assumed to be inactive, with no NCI compound being present in the WADA dataset after an initial screen that compared canonical SMILES strings.

**Table 1:** WADA class & number of molecules

WADA Class	Number of Molecules
P2	239
S1	47
S2	272
S3	367
S4	928
S5	1,000
S6	804
S7	195
S8	1,000
S9	26
Allowed	367
Total	5,245

### Conformer generation

Based on the original work of Ballester and Richards [20], only one low energy conformation per molecule was used. They showed, by taking one query molecule and generating 292 additional conformations, that the changes in the results from their Ultrafast Shape Recognition as a function of conformer were negligible. It has been shown [30] that taking more conformers into account can improve performance. However, to retain the descriptor's Ultrafast property, only the CORINA [27] generated conformation has been used.

### Descriptor

#### MACCS key descriptor

The MACCS key 166 bit descriptor was originally created by Molecular Design Limited (MDL) [11], and is a two dimensional substructure descriptor which encodes atoms, atom types, rings and bond information about the molecule. In this work, the MACCS key descriptor has been calculated using the Molecular Operating Environment software [21].

#### Ultrafast shape descriptor

Ballester and Richards' Ultrafast Shape Recognition descriptor [20] is a three dimensional descriptor that encodes the shape of the molecule based on the moments (mean, variance and skewness) of the distributions of interatomic distances. The fundamental principle underlying this descriptor is that the shape of a molecule is determined by the relative position of the atoms. The shape of a molecule can then be characterised using one-dimensional distributions, which encode three-dimensional information. This makes the descriptor quick to calculate, hence its name. Another advantage of this method is that it avoids the need for alignment or translation, as the distributions are independent of orientation or position. In their work, Ballester and Richards used distributions of atomic distances relative to four different reference points: the molecular centroid (ctd), the closest atom to the centroid (cst), the farthest atom from the centroid (fct) and the farthest atom from the farthest atom from the centroid (ftf), based on Euclidean distance. One of the problems initially found with this method was how to compare molecules with different numbers of atoms. The problem was solved by defining a fixed number of moments of the one dimensional distributions. In Ballester and Richards' work, they took the first three moments.

For example, using the centroid as the reference point, the first central moment is the average atomic distance to the molecular centroid, and provides a measure of the molecular size. The second central moment is the variance of the atomic distances about the centroid. The third central moment is the skewness of these atomic distances about the centroid and gives a measure of the asymmetry of the distribution. When calculated, this leads to a descriptor with 12 components, each component represents a floating point number calculated from a reference point and a moment; Ballester and Richards used four reference points and three moments. The Ultrafast descriptor with four central moments (UF4) has a length of 16 components.

In this work we have extended Ballester and Richards' Ultrafast shape recognition descriptor to include the fourth central moment (kurtosis *K*) [31] of the atomic coordinate distance distribution:

$$K=\frac{n\sum_{i=1}^{n}{\left(x_{i}-\mu \right)}^{4}}{(\sum_{i=1}^{n}{\left(x_{i}-\mu \right)}^{2}{)}^{2}}-3.$$

**Figure 1:**
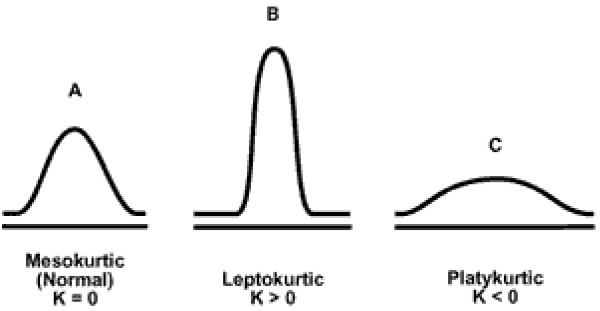
Mesokurtic, leptokurtic and platykurtic.

The kurtosis *K *is a measure of the peakedness of the distribution (see Figure 1).

Higher kurtosis means more of the variance is due to less frequent but more extreme deviations, as opposed to a lower value, indicating more frequent smaller deviations. The first five central moments (UF5) have also been calculated, to see if the fifth central moment improves the accuracy of the descriptor representation. The time taken to calculate the USR, UF4 and UF5 descriptors over the full data set is also considered.

### Classification

In this study, the Random Forest algorithm in R [32] has been used for multi-class classification. Ten multi-class classifications have been run. In each run a training, test and validation set have been used. The training set is composed of 50% of each prohibited class (P2 and S1-S9) and an equivalent number of inactive NCI compounds. For example, referring to the list of WADA classes (Table 1), 120 P2 compounds would have been selected in the training set in addition to 24 S1 compounds, 136 S2 compounds and so forth for all other classes, totaling 2,441 of the 5,245 WADA prohibited substances. The inactive training set was composed of 50% of the explicitly allowed WADA substances (184 compounds) and 2,257 NCI molecules giving a total of 2,441 inactives in the training set. The test and validation sets are made up of half of the prohibited substances (1,402) not used in the training set and half of the remaining inactives (55,487). The Random Forest models were built on the training set and applied in turn to the internal test set and the external validation set. Even though Random Forest does not need an internal test set for training, as do other methods such as an artificial neural network, we use an internal test set to adjust a threshold to optimise the classification in terms of the Matthews Correlation Coefficient, see below. All training, test and validation sets were constructed using random uniform sampling.

Random Forest is a machine learning algorithm based upon an ensemble of decision trees and was invented by Leo Breiman and Adele Cutler [33]. Each tree is grown by taking a bootstrap sample of N objects chosen at random *with replacement *from a training set containing N objects, so that the same object may appear more than once in the sample. For each node in the tree, a number *mtry *of descriptors is selected randomly. At each node, one of the *mtry *available descriptors is used for branch splitting, the descriptor chosen being that which yields the best active/inactive split at the node, based on the Gini Index. Each tree is then grown to its maximum extent with no pruning. When the test data are presented to the Random Forest, each tree's prediction is taken as an independent vote and the overall classification as active or inactive is determined according to the majority of these votes. We take the proportion of trees voting that a given molecule should be classified as active as an approximate measure of the probability of the molecule being active. Some of the advantages of Random Forest in a virtual screening setting are: efficient processing of large numbers of examples; capability to handle many input variables; estimate variable importance; by design immune to overfitting and problems due to missing data; and it can predict balanced class populations from unbalanced data sets. We note that some of these capabilities, especially though the simultaneous use of large datasets with a large number of variables, depend on the particular implementation.

We have trained a Random Forest classifier with 500 trees and a default *mtry *value of the square root of the number of descriptors for each training set rounded down (for example, a value of *mtry *= 12 would be used for the MACCS descriptor which has 166 bits, $\sqrt{166}=12.88$, which rounds down to 12). The model was then used to predict the probabilities that each individual molecule in the test and validation sets has come from each of the 10 prohibited activity classes or from the inactive class. The probability was estimated based on the fraction of trees voting for each specific class membership for each test and validation set molecule. The test and validation sets were then each ranked based on the probability of being part of each specific banned class; this resulted in ten class-specific ranked lists for each test and validation set per Random Forest run. This procedure was repeated for all four descriptors: MACCS, USR, UF4 and UF5. The USR and UF4 shape descriptors were then each separately combined with MACCS to form Hybrid descriptors and thus to see if the addition of extra three dimensional information would improve the virtual screening performance.

#### Threshold optimisation

A threshold was applied to each ranked internal test set list of probabilities to find the optimum cut-off probability that maximised the Matthews Correlation Coefficient with respect to that test set. The optimised threshold obtained from the test set was applied to the corresponding validation set (*i.e*., the threshold obtained for P2 test set fold 1 was applied to P2 validation set fold 1). Previous work [34] using the naive Bayes classifier has shown that optimising the threshold probability score based on the Matthews Correlation Coefficient can lead to better virtual screening performance. The thresholds optimised on the internal test set were used for predicting the activity classes of the molecules in the external validation set.

### Performance measures

In order to assess the accuracy of the classification and the effectiveness of the virtual screen, a number of performance measures have been used, namely: area under the Receiver Operating Characteristic curve (AUC), recall in the top 1% and top 5%, precision, Matthews Correlation Coefficient (MCC) and the F-measure of each ranked validation set.

$$Recall=\frac{t_{p}}{t_{p}+f_{n}}$$  X 100%

$$\mathrm{Precision}=\frac{t_{p}}{t_{p}+f_{p}}\;F-\mathrm{measure}=\frac{2*\mathrm{Recall}*\mathrm{Precision}}{\mathrm{Recall}+\mathrm{Precision}}$$

*t*_*p *_is the number of true positives, that is molecules of a particular activity correctly classified as exhibiting that activity. *t*_*n *_represents inactive molecules correctly predicted. *f*_*p *_and *f*_*n *_values represent the number of molecules incorrectly predicted to be active and inactive respectively.

The Matthews Correlation Coefficient [35,36] is defined by:

$$MCC=\frac{t_{p}t_{n}-f_{p}f_{n}}{\sqrt{\left(t_{p}+f_{p})(t_{p}+f_{n})(t_{n}+f_{p})(t_{n}+f_{n}\right)}}$$.

#### Area under the receiver operating characteristic curve

We have calculated the area under the ROC curve for each activity class in the validation sets and repeated this for each of the ten Random Forest runs, giving a total of 100 AUC values (10 runs, 10 activity classes). AUC is one of the most commonly used measures in the machine learning community, and can be interpreted as the probability that a classifier will assign a higher score to a positive example than a negative example if one from each class were picked at random.

#### Computational time

The wall clock time measures the time in seconds required to calculate shape descriptors for the 116,476 molecules used in this work.

#### Standard error of the mean

The standard error (S.E) of the mean

$$S.E=\frac{\hat{\sigma }}{\sqrt{n}}$$,

with $\hat{\sigma }$ the standard deviation of *n *independent runs, has been calculated over the ten Random Forest runs for the different descriptor methods, WADA prohibited classes and all performance measures.

## Results and discussion

### MACCS key descriptor

We find that the MACCS key descriptor is able to recall a large percentage of each WADA prohibited class in the top 1% and top 5% of the ranked validation sets, with values as high as 96% for the P2 class for the recall in the top 1%, see Table 2. The precision values are fairly high across the board with values ranging from 0.53 for the S8 class to 0.93 for P2. The MACCS key descriptor, however, performs poorly for the S1 and S9 classes. In the case of S1, a large number of false positives are predicted, giving a poor precision value of 0.07. The precision obtained for S9 using MACCS is also disappointing, with MACCS keys failing to predict true positives, as illustrated by the low recall values in the top 1% and top 5% of the ranked validation sets. The lowest of the AUC values is found to be 0.55 for S1, with intermediate values for other classes, and up to practically unity (perfect case scenario) for the P2 class. It should be noted that all AUC values have been rounded to two decimal places, and that the values for the P2 class are slightly below 1.00. The F-measure and Matthews Correlation Coefficient values are all moderately high, with six of the classes returning values greater than 0.6. Standard error results averaged over the ten runs are detailed in Table 3. All values are low, indicating consistency between the runs.

**Table 2:** Performance measures. Percentage of actives recalled in the top 1% and top 5% of the ranked validation sets, precision of predicted positives, area under the Receiver Operating Characteristic curve, F-measure and the Matthews Correlation Coefficient. All results are calculated over ten different runs and are based on the validation sets.

	Descriptor	P2	S1	S2	S3	S4	S5	S6	S7	S8	S9	Average
Recall 1%	Hybrid	100.00	76.36	90.29	94.73	85.86	57.60	91.74	88.96	40.92	55.00	78.15
	MACCS	96.17	50.83	51.03	43.08	82.49	63.96	90.50	80.41	41.88	0.00	60.04
	USR	34.58	70.91	51.03	43.08	46.98	13.28	32.39	25.00	8.76	6.67	33.27
	UF4	42.88	70.00	62.94	52.53	54.05	16.72	30.80	27.87	8.40	16.67	38.29
	UF5	69.06	41.45	32.17	25.59	15.78	4.07	12.34	21.05	3.22	0.00	22.47

Recall 5%	Hybrid	100.00	85.45	95.44	97.47	95.82	78.12	96.37	93.13	69.80	63.33	87.49
	MACCS	96.67	59.17	76.47	59.45	93.27	80.40	94.63	85.92	65.64	4.17	71.58
	USR	61.19	80.91	76.47	59.45	69.87	32.72	54.88	45.42	28.56	23.33	53.28
	UF4	73.39	77.27	83.82	65.82	74.01	38.76	53.98	42.19	29.04	41.67	58.00
	UF5	84.21	49.35	48.20	34.93	24.55	11.39	22.41	38.30	11.39	3.75	32.85

Precision	Hybrid	0.94	0.80	0.91	0.80	0.79	0.62	0.90	0.78	0.58	0.42	0.75
	MACCS	0.93	0.07	0.87	0.77	0.67	0.62	0.89	0.71	0.53	0.00	0.61
	USR	0.12	0.55	0.23	0.38	0.50	0.13	0.36	0.41	0.03	0.00	0.27
	UF4	0.32	0.69	0.17	0.51	0.71	0.08	0.51	0.49	0.27	0.00	0.38
	UF5	0.21	0.52	0.37	0.44	0.64	0.16	0.49	0.27	0.04	0.00	0.32

AUC	Hybrid	1.00	0.89	0.96	0.87	0.79	0.67	0.94	0.90	0.70	0.68	0.84
	MACCS	1.00	0.55	0.96	0.75	0.67	0.75	0.91	0.89	0.74	0.83	0.81
	USR	1.00	0.69	0.65	0.61	0.58	0.54	0.56	0.59	0.54	0.57	0.63
	UF4	1.00	0.77	0.64	0.72	0.60	0.55	0.57	0.63	0.53	0.55	0.66
	UF5	1.00	0.94	0.84	0.79	0.81	0.71	0.76	0.76	0.71	0.53	0.78

F-measure	Hybrid	0.91	0.54	0.82	0.72	0.71	0.45	0.83	0.70	0.27	0.22	0.62
	MACCS	0.91	0.11	0.79	0.67	0.63	0.51	0.84	0.63	0.29	0.00	0.54
	USR	0.11	0.27	0.17	0.23	0.35	0.06	0.22	0.08	0.04	0.00	0.15
	UF4	0.16	0.49	0.23	0.36	0.42	0.07	0.19	0.09	0.03	0.00	0.20
	UF5	0.10	0.31	0.20	0.23	0.36	0.07	0.22	0.07	0.03	0.00	0.16

MCC	Hybrid	0.91	0.58	0.83	0.73	0.71	0.47	0.83	0.71	0.32	0.26	0.63
	MACCS	0.91	0.13	0.79	0.68	0.63	0.52	0.84	0.64	0.32	0.00	0.55
	USR	0.12	0.32	0.18	0.25	0.37	0.08	0.24	0.13	0.08	0.00	0.18
	UF4	0.19	0.51	0.25	0.38	0.46	0.09	0.24	0.15	0.08	0.02	0.24
	UF5	0.13	0.34	0.23	0.26	0.40	0.09	0.26	0.10	0.06	0.00	0.19

**Table 3:** The standard error of the mean. Percentage of actives recalled in the top 1% and top 5% of the ranked validation sets, precision of predicted positives, area under the Receiver Operating Characteristic curve, F-measure and the Matthews Correlation Coefficient. All results are calculated over ten different runs and are based on the validation sets. The standard error values at the 95% confidence level were calculated over the ten different runs and ten classes.

	Descriptor	P2	S1	S2	S3	S4	S5	S6	S7	S8	S9	SE 95%
Recall 1%	Hybrid	0.000	3.090	1.319	1.020	0.812	1.010	0.566	1.585	0.962	4.339	3.924
	MACCS	0.255	1.944	3.142	1.092	4.183	1.000	0.381	1.330	4.379	0.000	5.679
	USR	2.652	4.242	1.612	1.369	0.972	0.710	0.837	2.106	0.665	2.722	4.025
	UF4	2.115	5.080	1.934	1.482	0.764	0.616	0.765	1.374	0.637	5.556	4.171
	UF5	0.015	0.018	0.017	0.015	0.005	0.002	0.006	0.011	0.002	0.000	4.131

Recall 5%	Hybrid	0.000	2.010	0.774	0.614	0.450	0.949	0.386	1.164	0.794	3.333	2.520
	MACCS	0.000	2.307	1.373	0.442	2.836	0.912	0.220	1.037	6.719	2.668	5.395
	USR	1.722	3.442	1.886	1.231	1.403	0.749	1.169	1.195	0.966	6.667	3.959
	UF4	1.805	3.105	1.404	1.609	0.733	0.917	1.023	2.633	1.040	5.693	3.772
	UF5	0.012	0.027	0.010	0.019	0.004	0.003	0.007	0.016	0.003	0.027	4.677

Precision	Hybrid	0.004	0.020	0.059	0.099	0.005	0.009	0.034	0.009	0.012	0.037	0.033
	MACCS	0.010	0.014	0.018	0.043	0.026	0.022	0.008	0.030	0.036	0.000	0.064
	USR	0.005	0.034	0.015	0.018	0.010	0.015	0.013	0.033	0.002	0.000	0.038
	UF4	0.017	0.017	0.007	0.019	0.009	0.005	0.020	0.033	0.028	0.000	0.048
	UF5	0.066	0.104	0.056	0.061	0.022	0.028	0.035	0.068	0.015	0.000	0.042

AUC	Hybrid	0.000	0.009	0.002	0.005	0.003	0.002	0.001	0.003	0.004	0.019	0.024
	MACCS	0.000	0.002	0.002	0.004	0.002	0.002	0.001	0.003	0.003	0.015	0.027
	USR	0.000	0.011	0.004	0.003	0.001	0.001	0.001	0.006	0.001	0.011	0.027
	UF4	0.000	0.010	0.004	0.004	0.002	0.000	0.001	0.007	0.001	0.015	0.028
	UF5	0.000	0.011	0.008	0.013	0.004	0.004	0.006	0.028	0.007	0.025	0.025

F-measure	Hybrid	0.003	0.016	0.035	0.052	0.003	0.002	0.027	0.006	0.004	0.019	0.047
	MACCS	0.006	0.021	0.008	0.015	0.012	0.009	0.007	0.017	0.010	0.000	0.061
	USR	0.003	0.014	0.009	0.004	0.003	0.003	0.003	0.007	0.001	0.000	0.022
	UF4	0.006	0.015	0.006	0.010	0.003	0.003	0.003	0.006	0.001	0.001	0.033
	UF5	0.016	0.059	0.020	0.019	0.016	0.008	0.013	0.019	0.006	0.000	0.024

MCC	Hybrid	0.914	0.580	0.828	0.727	0.711	0.468	0.830	0.708	0.316	0.257	0.044
	MACCS	0.006	0.025	0.008	0.015	0.011	0.009	0.007	0.016	0.010	0.000	0.060
	USR	0.116	0.315	0.183	0.252	0.372	0.081	0.237	0.130	0.075	0.001	0.023
	UF4	0.188	0.511	0.251	0.383	0.455	0.085	0.244	0.150	0.077	0.019	0.033
	UF5	0.019	0.064	0.013	0.018	0.010	0.007	0.011	0.025	0.010	0.001	0.026

### Shape descriptors

#### Computational time

It is not surprising that adding more moments increases the computational time required to calculate the descriptors for the 116,476 molecules in this dataset. The time taken for the USR descriptor to be calculated was 1,432 seconds, UF4 1,528 seconds and UF5 2,006 seconds. All results are based on the use of a 1.06 GHz Athlon processor and the implementation of the method in the Python programming language. Clearly the difference of less than 100 seconds is marginal between USR and UF4. However the computational time increases by an extra 478 seconds (31%) upon addition of the fifth central moment.

#### Performance

The results (Table 2) for USR, UF4 and UF5 as stand-alone methods are all fairly comparable and are worse than the MACCS key fingerprint on all measures. If one were to gauge performance based on recall in the top 1% of the ranked validation sets, the UF4 descriptor is the best method for 6 out of the 10 classes and in 6 out of 10 classes for the recall in the top 5%. With regards to precision, the UF5 descriptor is better than the USR descriptor for 7 of the classes, but only two classes (S2 and S5) when compared to UF4. The UF5 descriptor achieves the highest AUC values for 8 classes. When considering the F-measure, USR is worse than UF4 for 7 classes and UF5 is worse than UF4 for 6 classes. The Matthews Correlation Coefficient gives similar information to the F-measure and hence the results are very alike, with the UF5 being better than the USR for 7 out of the ten classes and better than the UF4 for only one class.

### Hybrid descriptor

In order to enhance the performance of the MACCS key descriptor we have combined it with UF4, the best performing shape descriptor based on computational performance and time to calculate, to form a Hybrid descriptor. The Hybrid descriptor combines the 166 bits of the MACCS key descriptor with the 12 components of the Ultrafast shape descriptor and four extra components from the fourth moment, to form a descriptor of length 182. The descriptor is composed of discrete (0s or 1s) data from the MACCS key descriptor and continuous data from the UF4 descriptor.

The results show a significant improvement in performance over MACCS when the Hybrid descriptor is used. This is particularly true for the values averaged over all classes, where the Hybrid descriptor is the best descriptor for all performance measures. The only exception was the S5 class, which consistently showed the MACCS key descriptor to give better results than the other methods, one possible explanation being that the molecules in the S5 classes have very diverse shapes resulting in the UF4 descriptor creating noise, hence the slight drop in performance relative to using MACCS on its own. MACCS was also combined with USR, this combination performing slightly worse than the MACCS-UF4 Hybrid descriptor we propose in this work. The standard error values for the 95% confidence level for the runs averaged over the ten runs and ten classes are detailed in Table 3, and support our finding that the Hybrid descriptor is the best descriptor. The Hybrid descriptor was found to be statistically significantly better than all other descriptors across all performance measures at the 95% confidence level.

## Conclusion

We have introduced a novel Hybrid descriptor for virtual screening of databases of chemical structures, which is quick to calculate, robust, and incorporates both two and three-dimensional information. The Hybrid descriptor gives better performance than either MACCS keys or the shape descriptors presented in this work based on: the recall in the top 1% and top 5% of the validation set, the positive precision, F-measure, Matthews Correlation Coefficient and the area under the Receiver Operating Characteristic curve. Incorporating an additional central moment, the kurtosis, into Ballester and Richards' [20] Ultrafast Shape Recognition descriptor, significantly improved its performance. The addition of the fifth central moment, however, does not improve the performance of UF4 sufficiently to justify the increased computational expense.

## Methods

### Ultrafast shape recognition

Ballester and Richards' USR descriptor and the UF4 shape descriptor were implemented in Python [37].

### Random Forest

The Random Forest classification algorithm was implemented in R [32].

## Authors' contributions

EOC wrote the first draft, developed the algorithms, and performed the experiments. FN and JBOM helped in the design of the experiment and the writing of the manuscript. All authors read and approved the final manuscript.

## Supplementary Material

Additional file 1 Breakdown of the WADA 2005 Dataset Generation. The table gives information on the breakdown of the composition of the WADA classes. Click here for file

Additional file 2 Most and Least Representative Structures (S1-S5). Information is given on the most and least representative structure for the S1, S2, S3, S4 and S5 WADA prohibited classes, based on the mean intra-class Euclidean distance using all 146 MOE 2D descriptors calculated from MOE 2004.03. Click here for file

Additional file 3 Most and Least Representative Structures (S6-P2). Information is given on the most and least representative structure for the S6, S7, S8, S9 and P2 WADA prohibited classes, based on the mean intra-class Euclidean distance using all 146 MOE 2D descriptors calculated from MOE 2004.03. Click here for file
